# Effect of Calcium and Manganese Supplementation on Heat Resistance of Spores of *Bacillus* Species Associated With Food Poisoning, Spoilage, and Fermentation

**DOI:** 10.3389/fmicb.2021.744953

**Published:** 2021-10-11

**Authors:** Martti Tapani Sinnelä, Alixander Mattay Pawluk, Young Hun Jin, Dabin Kim, Jae-Hyung Mah

**Affiliations:** Department of Food and Biotechnology, Korea University, Sejong, South Korea

**Keywords:** *Bacillus* species, *Bacillus* spores, heat resistance, calcium, manganese, mineral, spore starter culture

## Abstract

Bacterial spores often survive thermal processing used in the food industry, while heat treatment leads not only to a decrease in the nutritional and organoleptic properties of foods, but also to a delay in fermentation of fermented foods. Selective reduction of undesirable spores without such impediments is an ongoing challenge for food scientists. Thus, increased knowledge of the spore-forming bacteria is required to control them. In this study, the heat resistance results (*D*_100__°__C_) of the spores of four *Bacillus* species were determined and compared to previous literature, and found that *B. cereus* has significantly lower heat resistance than the other *Bacillus* species, *B. coagulans*, *B. subtilis*, and *B. licheniformis*. Using the spores of these strains, this study also evaluated the effects of single and combined supplementation of calcium (0.00–2.00 mM) and manganese (0.00–0.50 mM) on heat resistance (*D*_100__°__C_). The results revealed that the spores of *B. licheniformis* and *B. cereus* displayed the smallest heat resistance when sporulated on media rich in calcium. Conversely, *B. coagulans* spores and *B. subtilis* spores exhibited the greatest heat resistance when sporulated under calcium-rich conditions. The opposite results (stronger heat resistance for *B. licheniformis* spores and *B. cereus* spores, and smaller heat resistance for *B. coagulans* spores and *B. subtilis* spores) were obtained when the spores were formed on media poor in the minerals (particularly calcium). Based on the results, the *Bacillus* species were divided into two groups: *B. licheniformis* and *B. cereus*; and *B. coagulans* and *B. subtilis*. The study provides valuable insight to selectively reduce spores of undesirable *Bacillus* species in the food industry.

## Introduction

Spore-forming bacteria form a dormant, metabolically inert structure called a (endo) spore when encountering environmental stress factors such as nutrient exhaustion or starvation. The formed spores survive against disinfection and sterilization procedures as well as other adverse conditions (Nicholson et al., 2000; Ryu et al., 2005). Total elimination or inactivation of vegetative cells can be achieved easily, but spores may either stay unaffected by such treatments or only sustain low levels of damage. Furthermore, the application of conventional sterilization methods is often limited in food processing since high-intensity treatments can decrease the nutritional value and organoleptic characteristics of foods. Likewise, excessive heat treatment can reduce the number of beneficial bioactive microorganisms in fermented foods and/or delay the fermentation of foods (Mah et al., 2019), which limits the application of sterilization processes to the production of fermented food products. Hence, spores are usually considered inevitable contaminants in certain food products (Li et al., 2020). Due to this, eliminating or reducing spore contamination is one of the keys to guarantee the safety of foods such as fermented food products. Taken together, information on the heat resistance of spores in various environments is important for the development of a precise heat treatment process that minimizes both the risk of spores and the loss of nutrients in the food industry.

It is well known that many cultural and nutritional parameters, such as the mineral composition of the sporulation medium, have impacts on spore formation, although the sporulation abilities are predominantly genetically determined (Oomes and Brul, 2004; Sella et al., 2014). Incidentally, higher mineralization of sporulation medium is usually associated with higher levels of minerals in formed spores (especially in the core of spores) and decreased water content in the core (Bender and Marquis, 1985; Marquis and Bender, 1985; Nicholson et al., 2000). It is still somewhat unclear why the mineralization of the core significantly affects the heat resistance of spores (Sanchez-Salas et al., 2011). However, it has been empirically demonstrated, with several species of *Bacillus*, that elevated Ca^2+^ concentration in the spore core results in the greatest increase in heat resistance, whereas high levels of Mn^2+^ and Mg^2+^ slightly increase heat resistance, while spores formed with K^+^ and Na^+^ additions have the least heat resistance (Amaha and Ordal, 1957; Bender and Marquis, 1985; Marquis and Bender, 1985). The strong ability of calcium to enhance heat resistance of spores is closely related to pyridine-2,6-dicarboxylic acid (dipicolinic acid, DPA). Calcium can chelate DPA to form Ca-DPA, which efficiently reduces the water content in the spore core, thereby strengthening the spore structure, and even assisting in sporulation (Paidhungat et al., 2000; Cazemier et al., 2001; Setlow et al., 2006). The role of manganese in sporulation is regarded to be the most well-known among minerals (Igura et al., 2003; Stoeckel et al., 2009). Many authors have stated that manganese stimulates the growth and sporulation of various species of *Bacillus*: *B. megaterium* (Levinson and Hyatt, 1964; Verma et al., 2013), *B. subtilis* (Igura et al., 2003; Granger et al., 2011), *B. brevis* (Curran and Evans, 1954), *B. coagulans* (Amaha and Ordal, 1957), *B. cereus* (Ryu et al., 2005), and *B. fastidious* (Aoki and Slepecky, 1973). However, only a part of the aforementioned studies reported that manganese increased the heat resistance of the formed spores (Levinson and Hyatt, 1964; Aoki and Slepecky, 1973).

Though it is clear that bacterial spores provide resistance to moist heat (Gould, 2006; Coleman et al., 2007), much less is known about the difference in heat resistance between spores of different *Bacillus* species. Also, few attempts have been made with both calcium and manganese, including combinations thereof, to enhance or reduce the heat resistance of *Bacillus* spores. Therefore, the objective of the current study was to compare the heat resistance of spores of four representative *Bacillus* species associated with food poisoning and spoilage, as well as food fermentation, and to comparatively evaluate the influence of supplementation (no, single, and combination) of calcium and manganese on the heat resistance (viz., decimal reduction time).

## Materials and Methods

### *Bacillus* Strains

*Bacillus* strains tested in this study, including *B. licheniformis* (KCTC 1918), *B. cereus* (KCTC 3624), *B. coagulans* (KCTC 3625), and *B. subtilis* (KCTC 3135), are type strains and were obtained from the Korean Collection for Type Culture (KCTC; Daejeon, South Korea). The strains were grown in tryptic soy broth (TSB; Becton Dickinson, Sparks, MD, United States) for 24 h at the temperatures recommended by the KCTC (*B. licheniformis* and *B. coagulans* at 37°C, for *B. subtilis* and *B. cereus* at 30°C), and then kept as glycerol stock cultures (60%, v/v) in a freezer at −20°C.

### Comparison of Heat Resistance Data of Spores of Four *Bacillus* Species in Literature

To compare the heat resistance (*D*_10__0__°__C_-values) of spores of the four *Bacillus* species presented in the current and previous studies, literature was searched using search engines, including Web of Science and Google Scholar. Literature data compiled in Figure 2 and Supplementary Tables 1–4 were selected based on the following criteria: (1) minerals should not be added into the sporulation media in spore crop preparation and (2) *D*_100__°__C_-values should be obtained through heat treatment in a solution with a pH of approximately 7.0.

### Preparation of Cations-Supplemented Sporulation Media

Nutrient agar (NA; MB cell, Seoul, South Korea) served as the basal sporulation medium. Calcium chloride dihydrate (CaCl_2_⋅2H_2_O; Duchefa Biochemie, Harleem, Netherlands) and manganese sulfate monohydrate (MnSO_4_⋅H_2_O; Biosesang, Seoul, South Korea) acted as the cations added to NA. Diverse concentrations of Ca^2+^ (0.00, 0.25, 0.50, 1.00, and 2.00 mM) and Mn^2+^ (0.00, 0.10, 0.25, and 0.50 mM) were used to supplement the basal sporulation medium, similar to a previous report by Sinnelä et al. (2019) as based on earlier studies (Amaha and Ordal, 1957; Levinson and Hyatt, 1964). All sporulation media had a pH of 6.95–7.00, and prepared in Petri dishes with a diameter of 90 mm (SPL Life Sciences, Pocheon, South Korea).

### Spore Formation

**FIGURE 1:**
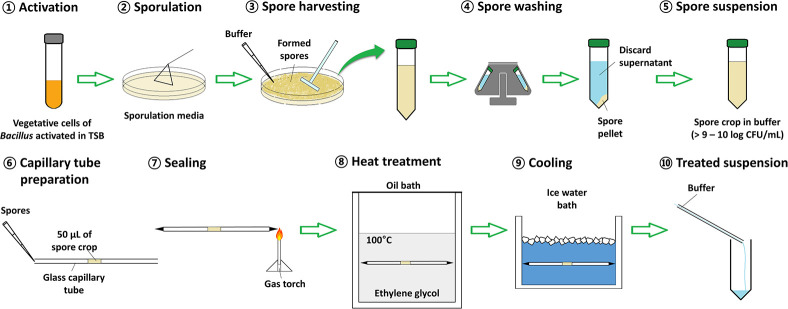
Scheme of the procedure for spore formation and heat treatment. 1: A single colony of *Bacillus* spp. was activated in 5 mL of tryptic soy broth at 30 or 37°C for 24 h. 2: Twenty plates of each sporulation medium (nutrient agar supplemented with a selected combination of minerals) were inoculated with 100 μL of *Bacillus* spp. culture and sporulated at 30 or 37°C for 48 h. 3: The formed spores were collected by pouring M/15 Sörensen’s phosphate buffer on the 20 agar plates, scraping the surfaces with a cell scraper, and transferred into a conical tube. 4: The spores were washed with the buffer by centrifuge at 10,000 × *g* for 10 min, 4°C, three times, intermittently discarding the supernatant. 5: The final spore crop was adjusted to a volume of 40 mL yielding a final spore concentration of >9–10 log CFU/mL. 6: A 50 μL aliquot of the spore crop was pipetting into the center of a glass capillary. 7: The capillary was sealed at both ends using a gas torch. 8: The loaded capillary was submerged in an oil bath set at 100°C. 9: At set time intervals, a capillary was removed and immediately placed in ice water to cool for 1 min. 10: The ends to the capillary were aseptically cut and the content was flushed into a conical tube using 3 mL of M/15 Sörensen’s phosphate buffer. The treated suspension was then serially diluted and plated on plate count agar.

The glycerol stocks of *Bacillus* strains were pre-activated before use by three successive subculturings in TSB for 24 h at the temperatures recommended for vegetative growth of respective strains described above. To form *Bacillus* spores, the sporulation media, described in the previous section, was inoculated with 0.1 mL of vegetative cell suspension by spreading, and the cells were sporulated for 2 days (Bai, 2013) at the temperatures for vegetative growth of respective *Bacillus* strains. Spores were then harvested by flooding the agar surface with M/15 Sörensen’s phosphate buffer (5.675 g of sodium phosphate dibasic and 3.63 g of potassium phosphate monobasic in 1 L of distilled water, pH 7.0; chemicals from Sigma-Aldrich Co., St. Louis, MO, United States) and scraping with a cell scraper (SPL Life Sciences). The spores from multiple plates were pooled, washed three times in a centrifuge (1736R; Gyrozen Co., Ltd., Daejeon, South Korea) at 19,000 × *g* for 10 min at 4°C, resuspended in the same phosphate buffer (to a final concentration of 9 log CFU/mL), and refrigerated at 4°C prior to being tested for heat resistance. The procedure for spore formation is illustrated in Figure 1 (step 1–5).

### Determination of Heat Resistance of Spores

The *D*-values (time required for a 10-fold reduction in viable spores) of *Bacillus* spores were determined by using the multiple-point method. Fifty microliters of spore suspension (prepared in section “Spore Formation”) were injected into a glass capillary tube with an inner diameter of 1.8 mm and an outer diameter of 3.0 mm (Corning Inc., Corning, NY, United States) using a pipette, and then both ends of the tubes were heat sealed. The tubes were fully immersed in a 100°C oil bath (Thermo Fisher Scientific Inc., Newington, NH, United States) filled with ethylene glycol (Samchun Pure Chemical Co., Ltd., Pyeongtaek, South Korea) set at 100 ± 0.015°C and heated for five different time intervals (*B. licheniformis*, 20 s intervals; *B. cereus*, 10 s intervals; *B. coagulans* and *B. subtilis*, 50 s intervals). After heating, the tubes were removed from the oil bath, cooled immediately in a crushed ice water bath, and washed with 70% ethyl alcohol. Both ends of the tubes were cut aseptically, and the content was flushed out with 3 mL of the phosphate buffer (Sinnelä et al., 2019). To enumerate spore survival, the treated suspension was 10-fold serially diluted in sterile 0.1% peptone water, and 0.1 mL of each dilution was spread onto plate count agar (PCA; Becton Dickinson) plates and incubated for 24 h at temperatures for vegetative growth of respective *Bacillus* strains described above. Following, the colonies of spore survivors were counted. The procedure for heat treatment of spores is illustrated in Figure 1 (step 6–10).

Survivor curves were plotted on a semi-log chart (log survivors versus time) to determine *D*-values. The *D*-values were obtained by taking the negative reciprocals of the slopes from linear regression of the survivor curves in Supplementary Figures 1–4.

### Statistical Analysis

**FIGURE 2:**
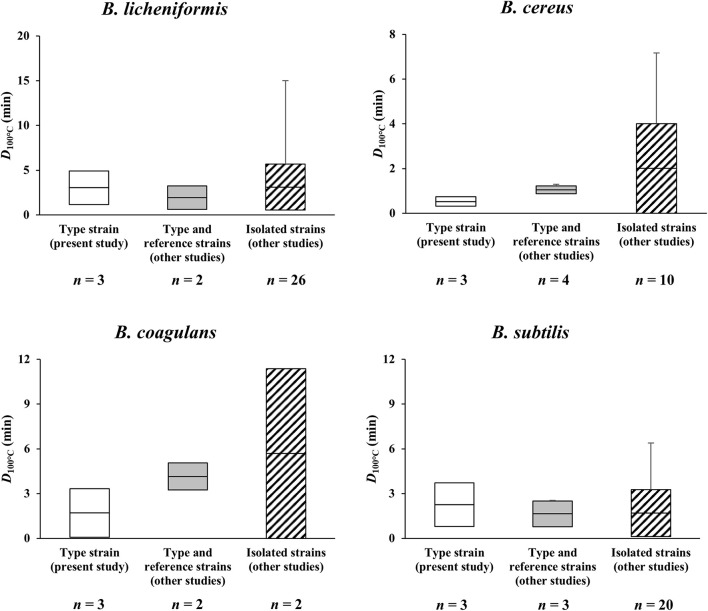
Overview of *D*_100__°__*C*_-values for spores of *B. licheniformis*, *B. cereus*, *B. coagulans*, and *B. subtilis* formed on basal media without mineral supplementation. The horizontal lines in the boxes indicate mean *D*_100__°__*C*_-values, and the boxes extend to the values of standard deviation after removal of outliers *via Q*-test. The error bars range from the minimum to the maximum *D*_100__°__*C*_-values. There were no statistical differences (*p* > 0.05) between mean *D*_100__°__*C*_-values included in the plot for each *Bacillus* species. 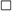: *D*_100__°__*C*_-values of the spores of type strains of *Bacillus* species determined in the present study, 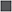: *D*_100__°__*C*_-values of the spores of type and reference strains obtained from previous studies, 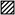: *D*_100__°__*C*_-values of the spores of isolated *Bacillus* strains obtained from previous studies. *D*_100__°__*C*_-values of each type strain used in the present study were determined from different spore batches, and *D*_100__°__*C*_-values obtained from previous studies were selected by the criteria described in section “Comparison of Heat Resistance Profiles of Spores of Four Representative *Bacillus* Species.” *n*: number of *D*_100__°__*C*_-values (measured independently) present in each bar.

The measurement of *D*_100__°__C_-values was conducted in triplicate, and the data were expressed as mean and standard deviation of the values obtained from a single spore batch. All values in Figure 2 were calculated from *D*_100__°__C_-values of different spore batches. Statistical analysis was performed by one-way analysis of variance (ANOVA) with Fisher’s pairwise comparison test. Differences were considered statistically significant at *p*-values of <0.05.

## Results and Discussion

### Comparison of Heat Resistance Profiles of Spores of Four Representative *Bacillus* Species

Heat resistance profiles of spores of *B. licheniformis*, *B. cereus*, *B. coagulans*, and *B. subtilis* formed on basal media without mineral supplementation were compiled in Figure 2 (also refer to Supplementary Tables 1–4). In the present study, the *D*_100__°__*C*_-values of the spores of the type strains were determined to be 3.91 ± 1.65 min for *B. licheniformis*, 0.49 ± 0.28 min for *B. cereus*, 1.70 ± 1.63 min for *B. coagulans*, and 2.73 ± 1.71 min for *B. subtilis*, which were similar to those of type (representing each species) and reference (serving as definitive control) strains examined in previous studies, respectively (*p* > 0.05). The reported *D*_100__°__*C*_-values were 1.94 ± 1.30 min for *B. licheniformis* (Nakayama et al., 1996; Esteban et al., 2015), 1.05 ± 0.17 min for *B. cereus* (Beaman et al., 1982; Rajkowski and Mikolajcik, 1987; Penna and Moraes, 2002; De Vries et al., 2004), 4.15 ± 0.91 min for *B. coagulans* (Nakayama et al., 1996; Majeed et al., 2021), and 1.64 ± 0.86 min for *B. subtilis* (Nakayama et al., 1996; Esteban et al., 2015). Note that type strains represent each species in taxonomic studies, and reference strains are used as definitive control strains for various microbiological testing procedures (National Collection of Type Cultures, 2015), regardless of the isolation sources. In some cases, type strains can be used as reference strains depending on the research purpose. It is also noteworthy that, for the above comparison, all literature data as much as possible were collected based on the following criteria: (1) minerals should not be added into the sporulation media in spore crop preparation and (2) *D*_100__°__*C*_-values should be obtained through heat treatment in a solution with a pH of approximately 7.0. The reasons for setting these criteria were to precisely compare the heat resistance profiles of spores of four *Bacillus* spp. to those of previous studies conducted under the same test conditions as the current study, and avoid influencing factors, such as strains tested, sporulation methods and media, suspension media, and heating methods (Den Besten et al., 2018). Based on the observation in the present study, mean *D*_100__°__C_-value of the spores of *B. licheniformis* calculated from the heat resistance is significantly higher than those of *B. subtilis* and *B. coagulans* (although the mean value for *B. subtilis* spores is higher than that of *B. coagulans* spores), while that of *B. cereus* is the lowest (*p* < 0.05). In literature, mean *D*_100__°__C_-value of the spores of *B. coagulans* is the highest, followed by *B. licheniformis*, *B. subtilis*, and *B. cereus* (although there were no statistical differences among the latter three *Bacillus* species). Such observation is expected to be important information that can be utilized to selectively reduce the spores of undesirable pathogenic *B. cereus*.

Meanwhile, in previous studies, the *D*_100__°__C_-values of the spores of the isolated *Bacillus* strains have been determined to be 3.11 ± 2.57 min for *B. licheniformis* (Janštová and Lukášová, 2001; André et al., 2013), 1.73 ± 2.00 min for *B. cereus* (Rajkowski and Mikolajcik, 1987; Janštová and Lukášová, 2001), 5.28 ± 5.69 min for *B. coagulans* (Janštová and Lukášová, 2001; André et al., 2013), and 1.70 ± 1.58 min for *B. subtilis* (Janštová and Lukášová, 2001; Movahedi and Waites, 2002; Conesa et al., 2003). The *D*_100__°__C_-values of the type and reference strains in the present and previous studies fall within the ranges of *D*_100__°__C_-values for the isolated strains (*p* > 0.05). Such wide ranges of *D*_100__°__C_-values for the isolated strains might be attributed to strain diversity, growth conditions, and the sources from which the strains were isolated. As type and reference strains have been cultured for a long time in the laboratory conditions preferable for growth, phenotypic characteristics of the strains might be stable (Fux et al., 2005), whereas isolated strains may be more adapted to the environment in which they are naturally found. Furthermore, not only are natural environments often more diverse and extreme than laboratory conditions (Fux et al., 2005), but strain diversity can also influence heat resistance properties (Carlin et al., 2006; Luu-Thi et al., 2014; Berendsen et al., 2015). Therefore, it seems inevitable that characteristics and resistances of spores of isolated strains differ from laboratory strains. Indeed, several studies reported that *Bacillus* strains isolated from some processed foods (particularly food products processed using heat treatment) had more heat resistance than type and reference strains (André et al., 2013; Den Besten et al., 2018). In addition, regarding the isolated strains, there was no statistically significant difference between the heat resistances of the spores of four *Bacillus* species, unlike the aforementioned type strains.

### Effect of Mineral Supplementation on Heat Resistance Profiles of Spores of *Bacillus licheniformis* and *Bacillus cereus*

Heat resistance profiles of *B. licheniformis* spores formed under different supplementation conditions were compiled in Table 1 (also refer to Supplementary Figure 5). *B. licheniformis* spores displayed the greatest heat resistance (*D*_100__°__C_-value of 5.07 ± 0.20 min) when sporulated on the non-supplemented media, whereas the smallest heat resistance (2.17 ± 0.04 min) of the spores was observed when sporulated on media supplemented with both a moderate level of calcium (1.00 mM) and the highest concentration of manganese (0.50 mM). Janštová and Lukášová (2001) reported that for spores of 21 *B. licheniformis* strains formed on NA media without mineral supplementation, the highest *D*_100__°__C_-value was found to be 5.06 min; conversely, Nakayama et al. (1996) reported a lower *D*_100__°__C_-value of 1.02 min when the spores were formed under the same non-supplementation condition. The former result was almost identical to the highest *D*_100__°__C_-value obtained in the present study from *B. licheniformis* spores formed under the same non-supplementation condition. Conversely, the latter result was somewhat lower than the lowest *D*_100__°__C_-value obtained in the current study from spores prepared with mineral supplementation. This difference may be due to the diversity of strains tested in the current and the previous research (Carlin et al., 2006; Luu-Thi et al., 2014; Berendsen et al., 2015).

**TABLE 1:** Statistical differences in heat resistance of *B. licheniformis* and *B. cereus* spores as affected by diverse concentrations of calcium and manganese.

**Calcium (mM)**	**Manganese (mM)**	***D*_100__°__C_-value (min)^a^**	
		***B. licheniformis***	***B. cereus***
0.00	0.00	5.07 ± 0.20^A,+^	0.29 ± 0.01^A,+^
	0.10	3.74 ± 0.21^DEF^	0.26 ± 0.01^BC^
	0.25	3.37 ± 0.24^FGH^	0.25 ± 0.01^BC^
	0.50	3.67 ± 0.12^DEFG^	0.26 ± 0.00^BC^
0.25	0.00	3.60 ± 0.38^DEFGH^	0.25 ± 0.00^BCD^
	0.10	2.36 ± 0.42^I^	0.26 ± 0.00^BC^
	0.25	3.79 ± 0.15^DE^	0.24 ± 0.00^CD^
	0.50	3.88 ± 0.15^D^	0.26 ± 0.01^B^
0.50	0.00	4.50 ± 0.29^B^	0.26 ± 0.01^B^
	0.10	3.41 ± 0.54^EFGH^	0.23 ± 0.00^DE^
	0.25	3.88 ± 0.13^DE^	0.22 ± 0.00^EF^
	0.50	3.30 ± 0.26^GH^	0.23 ± 0.00^DE^
1.00	0.00	3.80 ± 0.26^DE^	0.22 ± 0.01^EF^
	0.10	3.24 ± 0.15^H^	0.23 ± 0.00^DE^
	0.25	3.34 ± 0.13^FGH^	0.25 ± 0.01^BC^
	0.50	2.17 ± 0.04^I,^*	0.25 ± 0.00^BC^
2.00	0.00	4.03 ± 0.34^CD^	0.21 ± 0.01^F,^*
	0.10	3.30 ± 0.05^FGH^	0.27 ± 0.00^B^
	0.25	2.49 ± 0.11^I^	0.26 ± 0.01^BC^
	0.50	4.39 ± 0.16^BC^	0.26 ± 0.01^BC^

^*a*^Mean ± SD of independent experiments performed in triplicate.^*A*–*I*^Mean values in the same column that are not followed by the same letters are statistically different (*p* < 0.05); *the lowest *D*_100__°__*C*_-value obtained; ^+^the highest *D*_100__°__*C*_-value obtained.

Similar to *B. licheniformis* spores, the greatest heat resistance of *B. cereus* spores (0.29 ± 0.01 min) was achieved when sporulated on the non-supplemented media, as shown in Table 1 (also refer to Supplementary Figure 6). In contrast, *B. cereus* spores exhibited the smallest (0.21 ± 0.01 min) and the second smallest heat resistance (0.22 ± 0.01 min) when sporulated on media supplemented solely with calcium at a concentration of 2.00 and 1.00 mM, respectively. The media supplemented with lower levels of both calcium (0.50 mM) and manganese (0.25 mM) also resulted in a *D*_100__°__C_-value of 0.22 ± 0.00 min (another second smallest heat resistance). Although limited information is available about the effects of calcium and manganese on the heat resistance of *B. cereus* spores, *D*_100__°__C_-values, ranging from approximately 0.2–0.6 min, similar to the present study, have been obtained by multiple researchers (Gonzáles et al., 1997; Mazas et al., 1997a,b, 2009; González et al., 1999). On the other hand, Janštová and Lukášová (2001) reported much higher *D*_100__°__C_-values for *B. cereus* spores ranging from 0.71 to 1.48 min. The previous studies have shown that research methods, such as mineral (and other additives) supplementation, sporulation temperature, heating medium (and its pH and a_w_), and strain tested, could have a significant impact on the *D*-values of *B. cereus* spores.

As for both *B. licheniformis* and *B. cereus* spores formed on media supplemented only with a single mineral in this study, 0.50 mM calcium supplementation significantly enhanced the heat resistance of the spores compared to the other concentrations of calcium. Nevertheless, the *D*_100__°__C_-values of all the spores obtained on media supplemented only with calcium were lower than that of the spores sporulated on media without mineral supplementation. Unlike calcium, variation in the concentration of manganese as the sole mineral in the sporulation media insignificantly affected the heat resistance of spores of both *Bacillus* species. Thus, the heat resistance profiles of *B. licheniformis* spores and *B. cereus* spores sporulated on media supplemented with a single mineral were almost similar to each other, showing that a calcium concentration of 0.50 mM is the most effective in enhancing heat resistance. Moreover, both *B. licheniformis* spores and *B. cereus* spores exhibited the greatest heat resistance when formed under non-supplementation conditions, as described above.

Interestingly, when manganese was supplemented along with 0.50 mM calcium, the concentration of manganese seemed to adversely affect the heat resistance of spores of both *Bacillus* species. However, manganese supplementation alongside 1.00 mM calcium, affected the heat resistance of spores of the two species in opposite ways: the heat resistance of *B. licheniformis* spores was weakened as the manganese concentration increased, while that of *B. cereus* spores was enhanced. It is also noteworthy that the lowest *D*_100__°__C_-values of *B. cereus* spores for each concentration of manganese (except for non-supplementation of manganese) were observed when the calcium concentration was 0.50 mM, but *B. licheniformis* spores did not exhibit such a calcium concentration-specific decrease in heat resistance. Incidentally, note that the mineral supplementation at all levels resulted in a decrease in the heat resistance of both *B. licheniformis* spores and *B. cereus* spores as compared to non-supplementation conditions.

### Effect of Mineral Supplementation on Heat Resistance Profiles of Spores of *Bacillus coagulans* and *Bacillus subtilis*

As opposed to *B. licheniformis* spores and *B. cereus* spores, both *B. coagulans* spores and *B. subtilis* spores exhibited the greatest heat resistance when sporulated on media supplemented solely with the highest concentration of calcium (2.00 mM), as shown in Table 2 (also refer to Supplementary Figures 7, 8). Another greatest heat resistance of spores of both *Bacillus* species was also achieved when sporulated on media supplemented with moderate levels of both calcium (1.00 mM) and manganese (0.25 mM). The highest *D*_100__°__C_-values of *B. coagulans* spores formed under each supplementation condition were 5.76 ± 0.33 and 5.75 ± 0.31 min, respectively, and those of *B. subtilis* spores were 6.54 ± 0.26 and 6.54 ± 0.66 min, respectively. On the contrary, the smallest heat resistance of *B. coagulans* spores and *B. subtilis* spores was observed when sporulated on media supplemented solely with manganese at a concentration of 0.25 and 0.50 mM, respectively. The lowest *D*_100__°__C_-values of spores of the two species were 3.01 ± 0.23 and 2.83 ± 0.35 min, respectively. Nakayama et al. (1996) reported two *D*_100__°__C_-values of 4.6 and 4.79 min obtained from *B. coagulans* spores formed on NA media without mineral supplementation, which was almost identical to the *D*_100__°__C_-value of 4.51 ± 0.12 obtained from *B. coagulans* spores formed under the same supplementation condition in this study (Table 2). However, it is worth mentioning that a large difference in the *D*_100__°__C_-value of *B. coagulans* spores appeared when the spores were heat treated in different heating menstrua (Mallidis et al., 1990). In the previous study, the reported *D*_100__°__C_-values of *B. coagulans* spores were as follows: 4.15 min in tomato serum (pH 4.24), 4.9 min in citrate buffer (pH 4.5), and 21.0 min in phosphate buffer (pH 7). Meanwhile, Conesa et al. (2003) reported a *D*_100__°__C_-value of 6.5 min obtained from *B. subtilis* spores formed on PCA media without mineral supplementation, which was identical to the highest *D*_100__°__C_-value of *B. subtilis* spores in this study. In addition, it seems most likely that basal sporulation media have a significant impact on the *D*-values of *B. subtilis* spores as spores formed on NA media without mineral supplementation have been reported to range from 0.85 to 2.13 min (Nakayama et al., 1996; Janštová and Lukášová, 2001), which is much lower than the former study using non-supplemented PCA media. While in the present study, *B. subtilis* spores also displayed a lower *D*_100__°__C_-value of 3.95 ± 0.29 min when sporulated on NA media with no supplementation (Table 2).

**TABLE 2:** Statistical differences in heat resistance of *B. coagulans* and *B. subtilis* spores as affected by diverse concentrations of calcium and manganese.

**Calcium (mM)**	**Manganese (mM)**	***D*_100__°__C_-value (min)^a^**	
		***B. coagulans***	***B. subtilis***
0.00	0.00	4.51 ± 0.12^EFGH^	3.95 ± 0.29^FG^
	0.10	3.91 ± 0.14^HI^	3.64 ± 0.26^GH^
	0.25	3.01 ± 0.23^J,^*	2.94 ± 0.40^HIJ^
	0.50	3.79 ± 0.34^I^	2.83 ± 0.35^J,^*
0.25	0.00	4.67 ± 0.33^EFG^	4.43 ± 0.13^EF^
	0.10	4.69 ± 0.32^EFG^	4.18 ± 0.29^FG^
	0.25	4.31 ± 0.17^GHI^	2.98 ± 0.11^HIJ^
	0.50	4.76 ± 0.34^CDEFG^	2.91 ± 0.08^IJ^
0.50	0.00	5.08 ± 0.46^BCDE^	4.93 ± 0.47^DE^
	0.10	5.05 ± 0.24^BCDEF^	3.62 ± 0.08^GH^
	0.25	4.58 ± 0.11^EFG^	3.55 ± 0.21^GHI^
	0.50	3.89 ± 0.32^HI^	3.15 ± 0.35^HIJ^
1.00	0.00	5.38 ± 0.35^ABCD^	4.56 ± 0.32^EF^
	0.10	5.40 ± 0.00^AB^	5.57 ± 0.31^BCD^
	0.25	5.75 ± 0.31^BCDE^	6.54 ± 0.66^A,+^
	0.50	4.62 ± 0.44^EFG^	5.56 ± 0.69^CD^
2.00	0.00	5.76 ± 0.33^A,+^	6.54 ± 0.26^AB,+^
	0.10	5.42 ± 0.24^ABC^	5.64 ± 0.26^BC^
	0.25	4.76 ± 0.00^DEFG^	5.50 ± 0.27^CD^
	0.50	4.44 ± 0.28^FGHI^	5.46 ± 0.46^CD^

^*a*^Mean ± SD of independent experiments performed in triplicate.^*A*–*J*^Mean values in the same column that are not followed by the same letters are statistically different (*p* < 0.05); *the lowest *D*_100__°__*C*_-value obtained; ^+^the highest *D*_100__°__*C*_-value obtained.

As for both *B. coagulans* spores and *B. subtilis* spores formed on media supplemented with only a single mineral, the concentrations of calcium and manganese significantly affected the heat resistance of the spores in opposite ways. In detail, the heat resistance of spores of both *Bacillus* species was enhanced as the calcium concentration increased. On the contrary, with the exception of supplementation only with 0.50 mM manganese for *B. coagulans* spores, the heat resistance of the spores of the two species was weakened as the manganese concentration increased. Amaha and Ordal (1957) empirically revealed that both calcium and manganese significantly enhanced the heat resistance of *B. coagulans* spores. Olivier et al. (2012) also reported that mineral supplementation, consisting of calcium, magnesium, and manganese, resulted in an increase in the heat resistance of *B. coagulans* spores compared to that of the spores formed under non-supplementation conditions. The previous studies are in agreement with the results obtained from supplementation with calcium, but contrary to the results obtained from supplementation with manganese in the current study. In *B. subtilis*, calcium has been well known to increase the heat resistance of spores (Bender and Marquis, 1985; Marquis and Bender, 1985; Oomes et al., 2007). However, the literature provides little information on the effect of manganese as a single mineral on the heat resistance of *B. subtilis* spores.

Interestingly, the lowest *D*_100__°__C_-values of both *B. coagulans* spores and *B. subtilis* spores formed at each concentration of manganese were observed when calcium was not supplemented. With a few exceptions, the *D*_100__°__C_-values generally rose as the calcium concentration increased. The results indicated that calcium is necessarily required to enhance the heat resistance of spores of both *B. coagulans* and *B. subtilis*, which is in agreement with the results obtained from the supplementation with calcium as a single mineral. In contrast, spores of both *B. licheniformis* and *B. cereus* did not exhibit such calcium concentration-dependent heat resistance. It is also worth mentioning that, unlike calcium, increasing the concentration of manganese often adversely affected the heat resistance of both *B. coagulans* spores and *B. subtilis* spores formed at each concentration of calcium, with a few exceptions. This is in accordance with the results obtained from the supplementation with manganese as a single mineral.

Taken together, depending on the response of spores to supplementation, the *Bacillus* spp. were split into two distinguished heat resistance groups: *B. licheniformis* and *B. cereus*; and *B. coagulans* and *B. subtilis*. In both *B. licheniformis* and *B. cereus*, the greatest heat resistance of spores was achieved on media without mineral sporulation, which did not significantly affect the heat resistance of *B. coagulans* spores and *B. subtilis* spores. Alternatively, the heat resistance of spores of both *B. coagulans* and *B. subtilis* was enhanced when sporulated on media with high levels (1.00–2.00 mM) of calcium supplementation, but that of *B. cereus* was significantly lowered under supplementation solely with 2.00 mM calcium.

## Conclusion

Thermal processing used in the food industry is often insufficient in eliminating bacterial spores. Excessive heat treatments can result in a decrease in the nutritional values and organoleptic characteristics of foods. In the production of fermented foods, heat treatment can delay or prevent fermentation by reducing or eliminating beneficial bioactive (and/or fermenting) microorganisms. It is therefore of great interest to develop a precise heat treatment process, enabling such heat treatment to selectively eliminate or inactivate undesirable spores such as *B. cereus* spores and to selectively allow the survival and growth of beneficial microorganisms, particularly the spores thereof. The surviving spores may have the potential to serve as “spore starter cultures” for fermentation. The development of precise heat treatment processes to sterilize undesirable spores in foods without compromising the nutritional values and sensory qualities of the foods is also important.

The results of the present study indicated that, based on the heat resistance profiles, *B. cereus* has significantly lower heat resistance than the other *Bacillus* species. As for the effect of mineral supplementation, the *Bacillus* spp. were split into two distinguished heat resistance groups: *B. licheniformis* and *B. cereus*; and *B. coagulans* and *B. subtilis*. The results also showed that the smallest heat resistance of both *B. licheniformis* spores and *B. cereus* spores as well as the greatest heat resistance of both *B. coagulans* spores and *B. subtilis* spores were observed when sporulated on media rich in calcium (1.00–2.00 mM). Conversely, the greatest heat resistance of the former spores was achieved when formed under non-supplementation conditions, while reduced heat resistance of the latter spores was obtained when sporulated in the absence of calcium. Thus, it seems possible to enhance the heat resistance of beneficial *Bacillus* spores while lowering the heat resistance of undesirable *Bacillus* spores, which may, in turn, lead to the development of precise heat treatment processes. Consequently, this study may be useful in developing intervention measures to improve the quality and safety of food products, including fermented foods, by providing insight into the diversity of heat resistance of *Bacillus* spores associated with food poisoning, spoilage, and fermentation.

## Data Availability Statement

The original contributions presented in the study are included in the article/Supplementary Material, further inquiries can be directed to the corresponding author.

## Author Contributions

J-HM designed and supervised the study. MS, AP, YJ, and DK collected and analyzed the data. MS, AP, and YJ drafted the manuscript. AP, YJ, and J-HM finalized this manuscript. All authors contributed to the article and approved the submitted version.

## Conflict of Interest

The authors declare that the research was conducted in the absence of any commercial or financial relationships that could be construed as a potential conflict of interest.

## Publisher’s Note

All claims expressed in this article are solely those of the authors and do not necessarily represent those of their affiliated organizations, or those of the publisher, the editors and the reviewers. Any product that may be evaluated in this article, or claim that may be made by its manufacturer, is not guaranteed or endorsed by the publisher.

## Supplementary Material

Supplementary Figure 1 Linear regression of thermal survivor curves for *B. licheniformis* spores formed under diverse supplementation conditions. **(A)** Manganese 0.00 mM, **(B)** manganese 0.10 mM, **(C)** manganese 0.25 mM, and **(D)** manganese 0.50 mM. **—**: Calcium 0.00 mM, : Calcium 0.25 mM, : Calcium 0.50 mM, : Calcium 1.00 mM, : Calcium 2.00 mM. Each regression line was drawn based on survivor data obtained after treatment at 100°C for five different time intervals.

Supplementary Figure 2 Linear regression of thermal survivor curves for *B. cereus* spores formed under diverse supplementation conditions. **(A)** Manganese 0.00 mM, **(B)** manganese 0.10 mM, **(C)** manganese 0.25 mM, and **(D)** manganese 0.50 mM. **—**: Calcium 0.00 mM, : Calcium 0.25 mM, : Calcium 0.50 mM, : Calcium 1.00 mM, : Calcium 2.00 mM. Each regression line was drawn based on survivor data obtained after treatment at 100°C for five different time intervals.

Supplementary Figure 3 Linear regression of thermal survivor curves for *B. coagulans* spores formed under diverse supplementation conditions. **(A)** Manganese 0.00 mM, **(B)** manganese 0.10 mM, **(C)** manganese 0.25 mM, and **(D)** manganese 0.50 mM. **—**: Calcium 0.00 mM, : Calcium 0.25 mM, : Calcium 0.50 mM, : Calcium 1.00 mM, : Calcium 2.00 mM. Each regression line was drawn based on survivor data obtained after treatment at 100°C for five different time intervals.

Supplementary Figure 4 Linear regression of thermal survivor curves for *B. subtilis* spores formed under diverse supplementation conditions. **(A)** Manganese 0.00 mM, **(B)** manganese 0.10 mM, **(C)** manganese 0.25 mM, and **(D)** manganese 0.50 mM. **—**: Calcium 0.00 mM, : Calcium 0.25 mM, : Calcium 0.50 mM, : Calcium 1.00 mM, : Calcium 2.00 mM. Each regression line was drawn based on survivor data obtained after treatment at 100°C for five different time intervals.

Supplementary Figure 5 Heat resistance profiles of *B. licheniformis* spores formed under diverse supplementation conditions. Bars and error bars represent means and standard deviations, respectively, calculated from triplicate experiments. The gray and black bars indicate the lowest and highest *D*_100__°__*C*_-values, respectively.

Supplementary Figure 6 Heat resistance profiles of *B. cereus* spores formed under diverse supplementation conditions. Bars and error bars represent means and standard deviations, respectively, calculated from triplicate experiments. The gray and black bars indicate the lowest and highest *D*_100__°__*C*_-values, respectively.

Supplementary Figure 7 Heat resistance profiles of *B. coagulans* spores formed under diverse supplementation conditions. Bars and error bars represent means and standard deviations, respectively, calculated from triplicate experiments. The gray and black bars indicate the lowest and highest *D*_100__°__*C*_-values, respectively.

Supplementary Figure 8 Heat resistance profiles of *B. subtilis* spores formed under diverse supplementation conditions. Bars and error bars represent means and standard deviations, respectively, calculated from triplicate experiments. The gray and black bars indicate the lowest and highest *D*_100__°__*C*_-values, respectively.
