# Supplementary figures and images for: Effect of Calcium and Manganese Supplementation on Heat Resistance of Spores of Bacillus Species Associated With Food Poisoning, Spoilage, and Fermentation
Source: Front Microbiol. 2021 Oct 11;12:744953. doi: 10.3389/fmicb.2021.744953 (PMC8542979; doi:10.3389/fmicb.2021.744953)

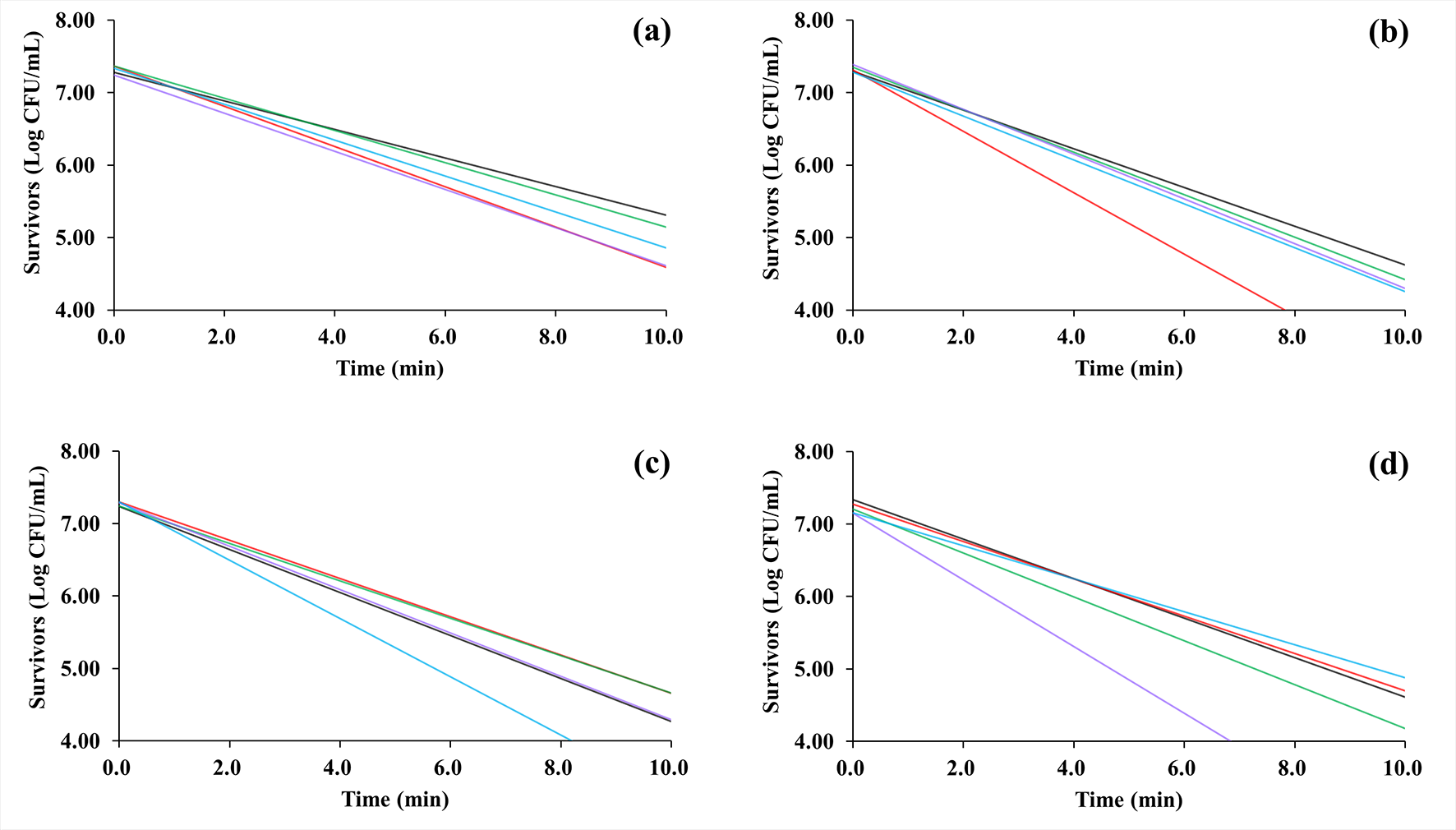

Supplement: Supplementary Figure 1 — Linear regression of thermal survivor curves for B. licheniformis spores formed under diverse supplementation conditions. (A) Manganese 0.00 mM, (B) manganese 0.10 mM, (C) manganese 0.25 mM, and (D) manganese 0.50 mM. —: Calcium 0.00 mM, : Calcium 0.25 mM, : Calcium 0.50 mM, : Calcium 1.00 mM, : Calcium 2.00 mM. Each regression line was drawn based on survivor data obtained after treatment at 100°C for five different time intervals. [file Image_1.TIF]

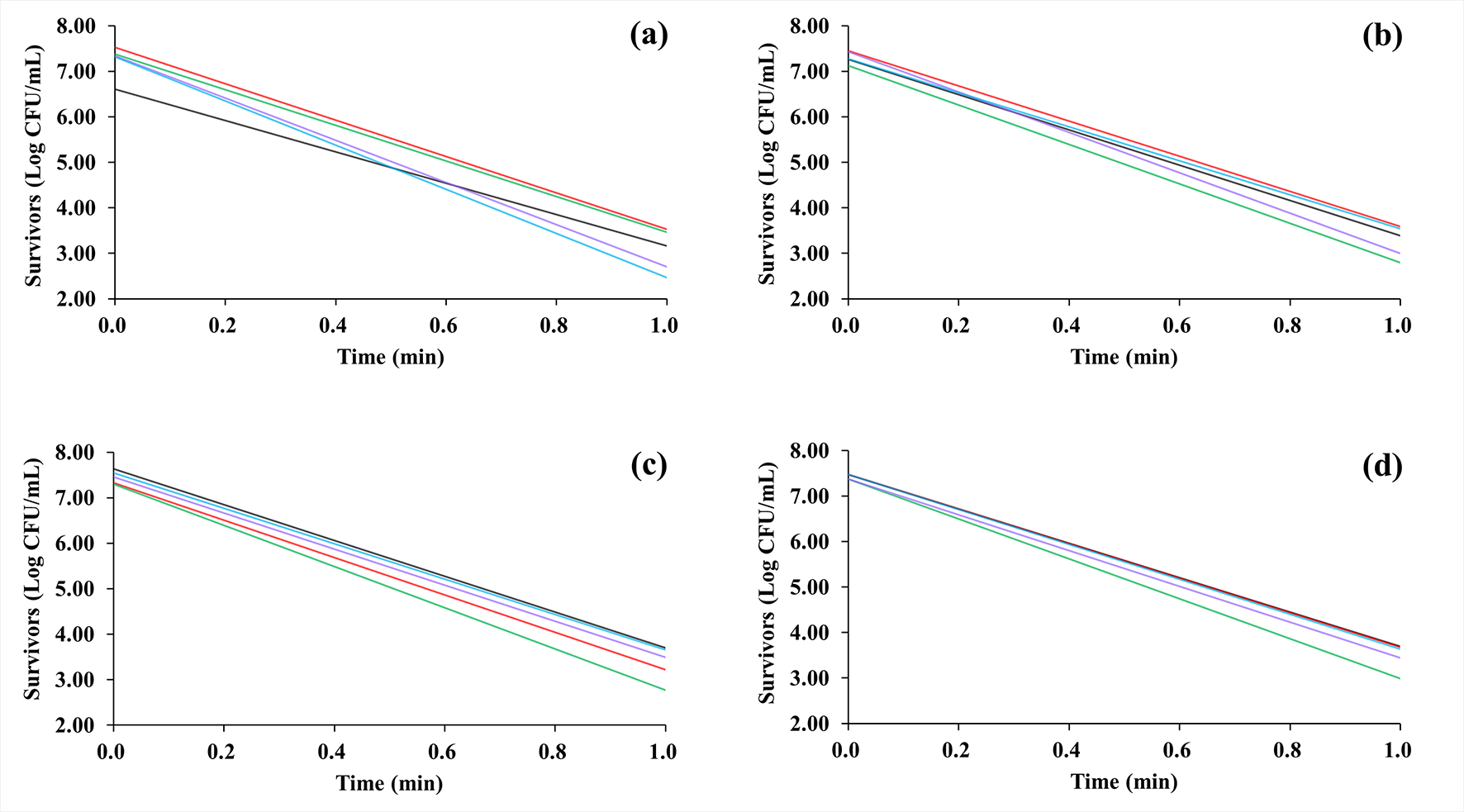

Supplement: Supplementary Figure 2 — Linear regression of thermal survivor curves for B. cereus spores formed under diverse supplementation conditions. (A) Manganese 0.00 mM, (B) manganese 0.10 mM, (C) manganese 0.25 mM, and (D) manganese 0.50 mM. —: Calcium 0.00 mM, : Calcium 0.25 mM, : Calcium 0.50 mM, : Calcium 1.00 mM, : Calcium 2.00 mM. Each regression line was drawn based on survivor data obtained after treatment at 100°C for five different time intervals. [file Image_2.TIF]

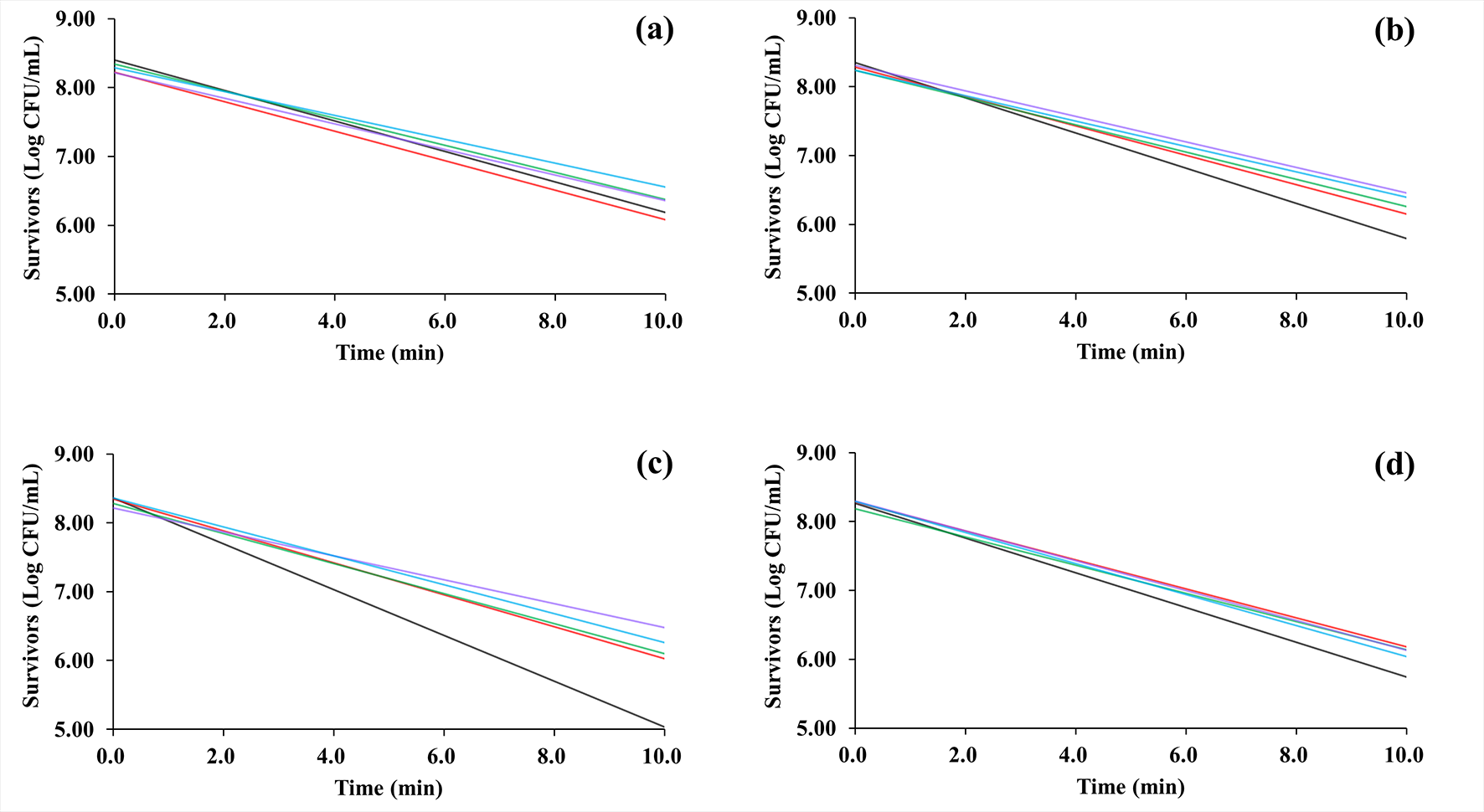

Supplement: Supplementary Figure 3 — Linear regression of thermal survivor curves for B. coagulans spores formed under diverse supplementation conditions. (A) Manganese 0.00 mM, (B) manganese 0.10 mM, (C) manganese 0.25 mM, and (D) manganese 0.50 mM. —: Calcium 0.00 mM, : Calcium 0.25 mM, : Calcium 0.50 mM, : Calcium 1.00 mM, : Calcium 2.00 mM. Each regression line was drawn based on survivor data obtained after treatment at 100°C for five different time intervals. [file Image_3.TIF]

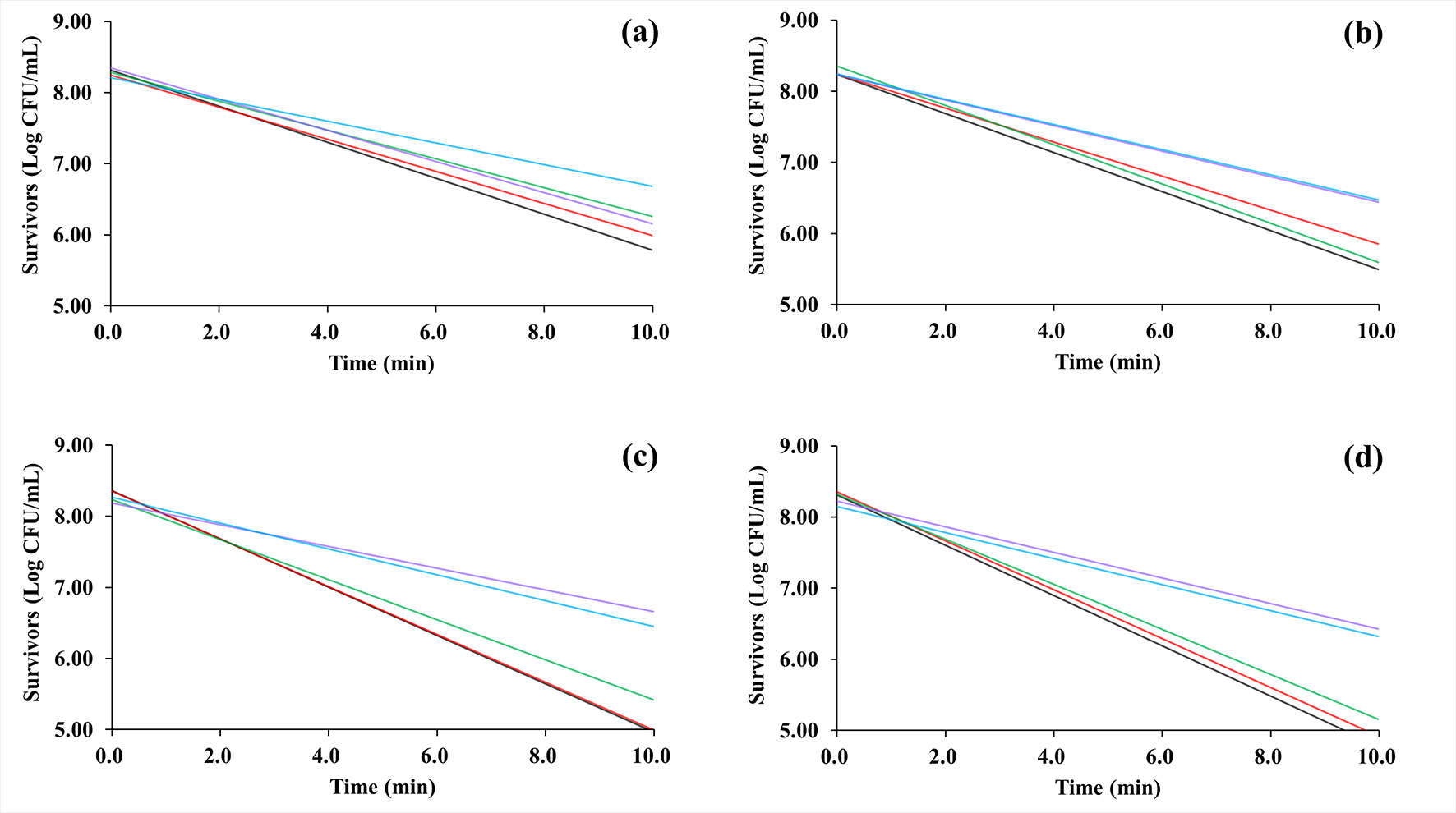

Supplement: Supplementary Figure 4 — Linear regression of thermal survivor curves for B. subtilis spores formed under diverse supplementation conditions. (A) Manganese 0.00 mM, (B) manganese 0.10 mM, (C) manganese 0.25 mM, and (D) manganese 0.50 mM. —: Calcium 0.00 mM, : Calcium 0.25 mM, : Calcium 0.50 mM, : Calcium 1.00 mM, : Calcium 2.00 mM. Each regression line was drawn based on survivor data obtained after treatment at 100°C for five different time intervals. [file Image_4.TIF]

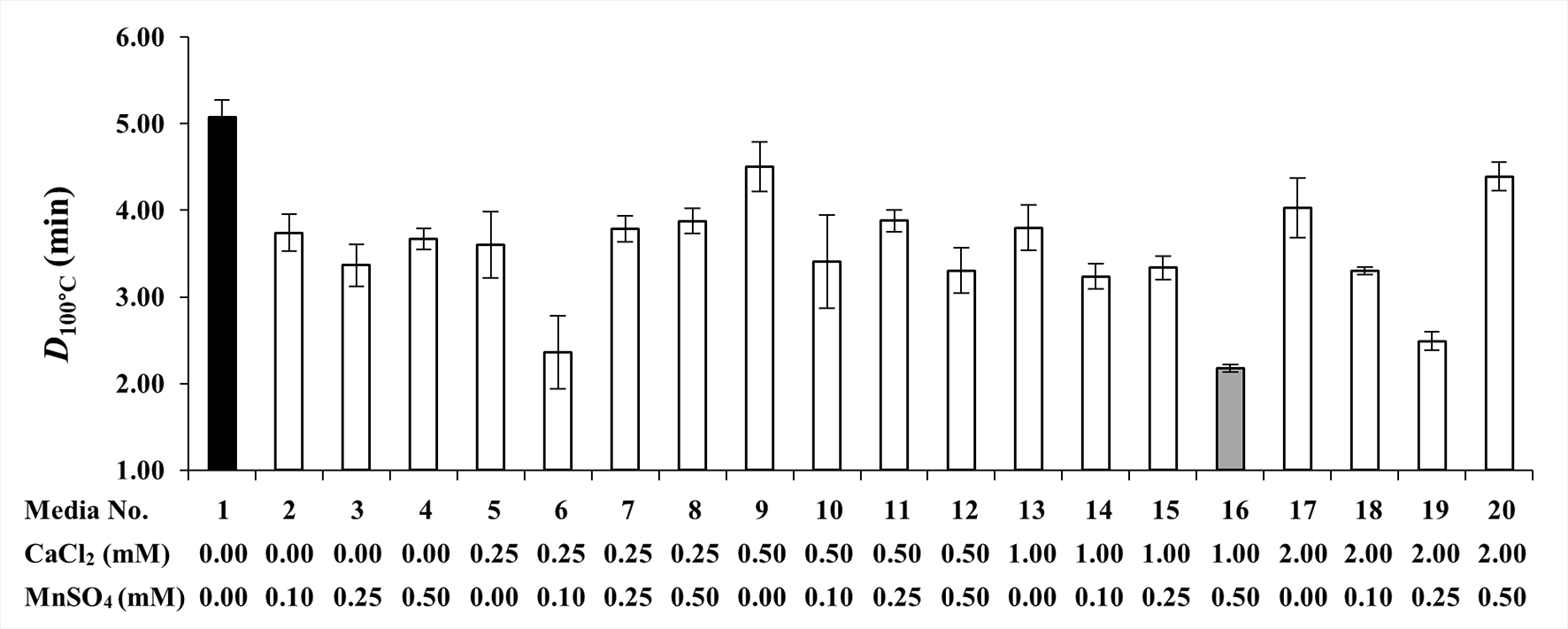

Supplement: Supplementary Figure 5 — Heat resistance profiles of B. licheniformis spores formed under diverse supplementation conditions. Bars and error bars represent means and standard deviations, respectively, calculated from triplicate experiments. The gray and black bars indicate the lowest and highest D100°C-values, respectively. [file Image_5.TIF]

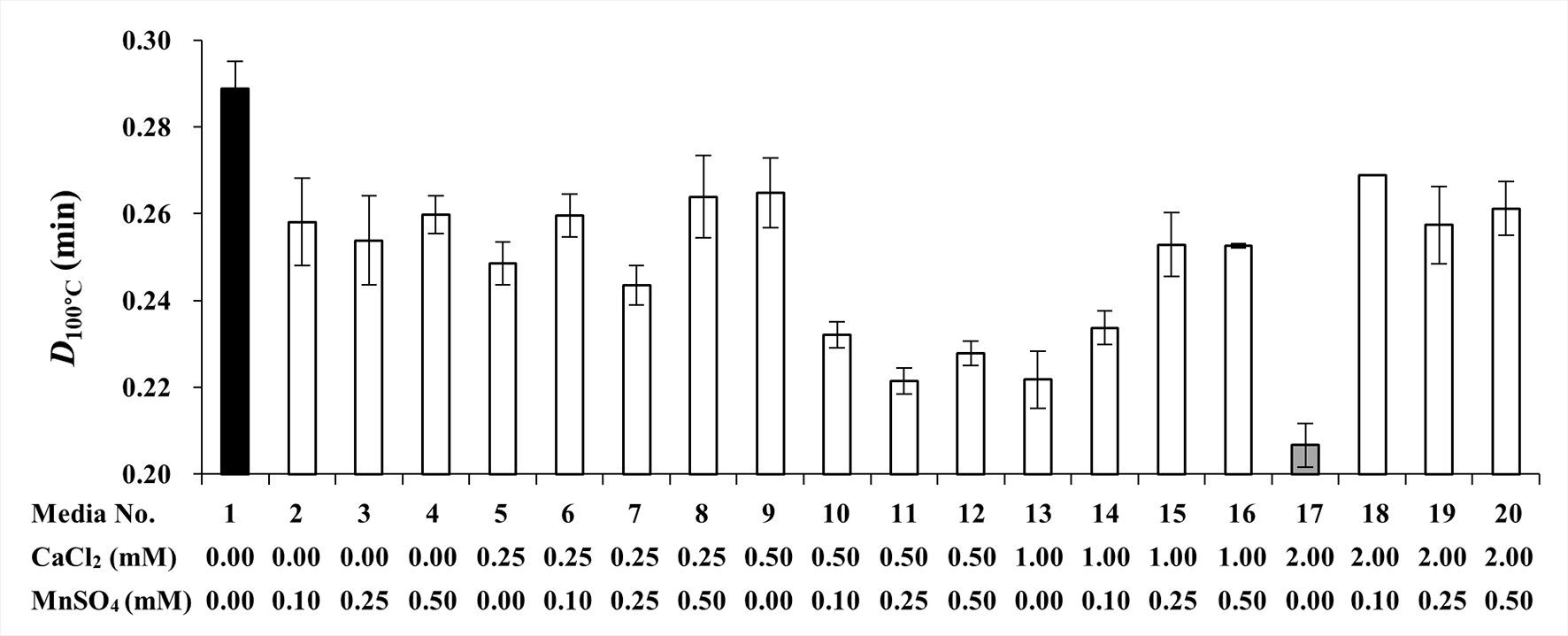

Supplement: Supplementary Figure 6 — Heat resistance profiles of B. cereus spores formed under diverse supplementation conditions. Bars and error bars represent means and standard deviations, respectively, calculated from triplicate experiments. The gray and black bars indicate the lowest and highest D100°C-values, respectively. [file Image_6.TIF]

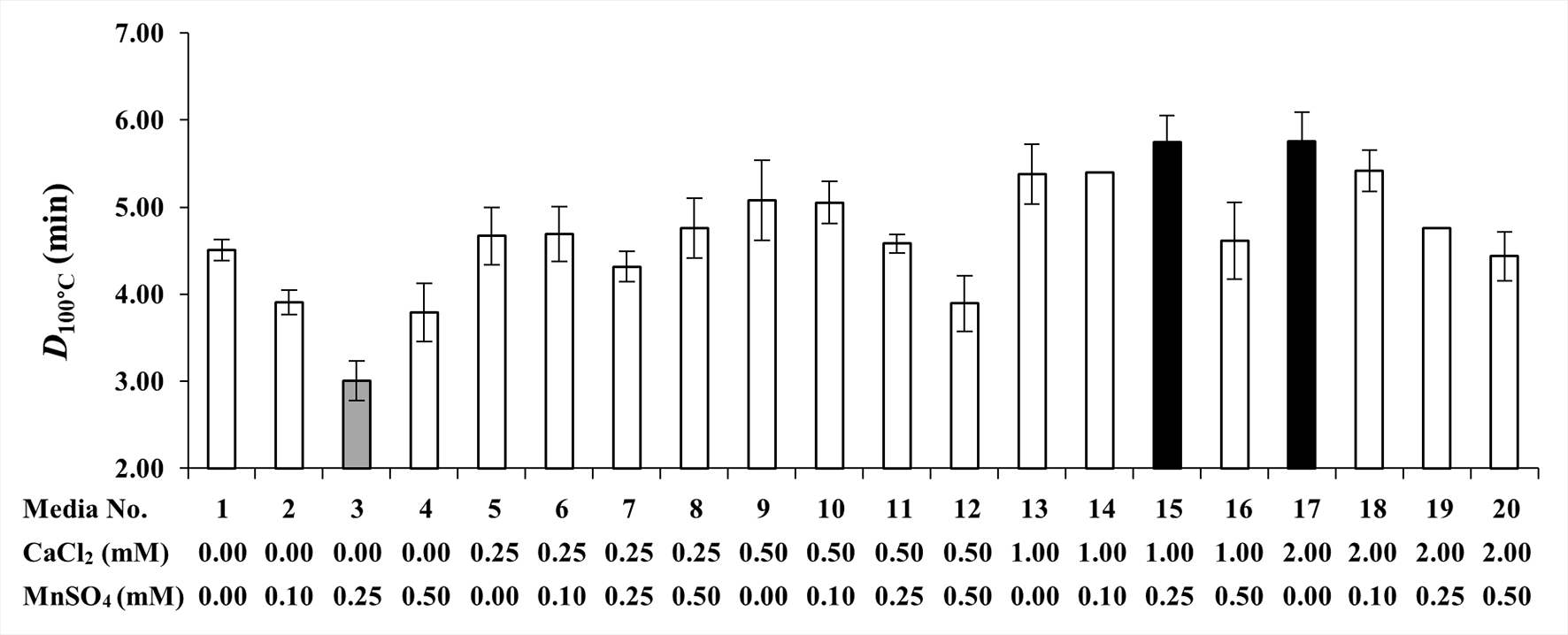

Supplement: Supplementary Figure 7 — Heat resistance profiles of B. coagulans spores formed under diverse supplementation conditions. Bars and error bars represent means and standard deviations, respectively, calculated from triplicate experiments. The gray and black bars indicate the lowest and highest D100°C-values, respectively. [file Image_7.TIF]

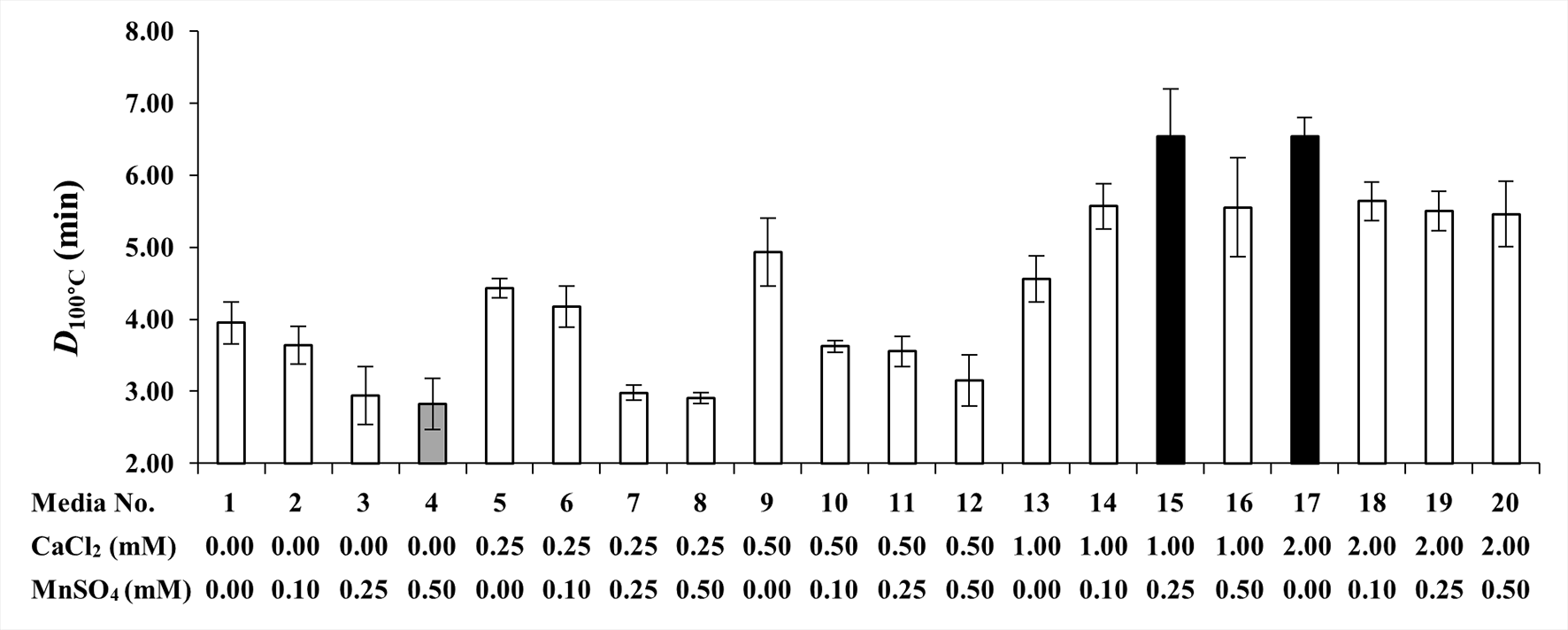

Supplement: Supplementary Figure 8 — Heat resistance profiles of B. subtilis spores formed under diverse supplementation conditions. Bars and error bars represent means and standard deviations, respectively, calculated from triplicate experiments. The gray and black bars indicate the lowest and highest D100°C-values, respectively. [file Image_8.TIF]
