# Supplementary material for: Effect of Calcium and Manganese Supplementation on Heat Resistance of Spores of Bacillus Species Associated With Food Poisoning, Spoilage, and Fermentation
Source: Front Microbiol. 2021 Oct 11;12:744953. doi: 10.3389/fmicb.2021.744953 (PMC8542979; doi:10.3389/fmicb.2021.744953)
Supplement: Supplementary file 9 [file Table_1.DOCX]

**Table S1.** Overview of *D*_100°C_-values for spores of *B. licheniformis* formed on basal media without mineral supplementation in literature

| Type of strains | Strains used | Number of strains | *D*_100°C_ (min) | Sporulation conditions | | |  | Heat treatment condition |  | Viable cell counting conditions | | | References |
| --- | --- | --- | --- | --- | --- | --- | --- | --- | --- | --- | --- | --- | --- |
|  |  |  |  | Media | Temperature (°C) | Time (h) |  | Suspension media |  | Media | Temperature (°C) | Time (h) |  |
| Type strain | IAM 13417 | 1 | 1.02 | Nutrient agar | 35 | 72 |  | Distilled water |  | Nutrient agar | 35 | 240 | Nakayama et al. (1996) |
| Reference strain | CECT 4523 | 1 | 2.86 | Plate count agar | 37 | NA ^a^ |  | Distilled water |  | Brain heart infusion agar | 37 | 24 | Esteban et al. (2015) |
| Isolated strain | NS ^b^ | 21 | 2.37 | Peptone water | 37 | 336 |  | Peptone water |  | Plate count agar | 37 | 24 | Janštová et al. (2001) |
|  | 3107 017 | 1 | 4.4 | Aerobic bacteria medium | 37 | 48-120 |  | 0.2 M phosphate buffer |  | Aerobic bacteria medium | 37 | 48-120 | André et al. (2013) |
|  | 3107 022 | 1 | 2.2 |  | 37 |  |  |  |  |  | 37 |  |  |
|  | 3107 028 | 1 | 15.2 |  | 37 |  |  |  |  |  | 37 |  |  |
|  | 3107 043 | 1 | 4.2 |  | 37 |  |  |  |  |  | 37 |  |  |
|  | 3107 043 | 1 | 5 |  | 55 |  |  |  |  |  | 55 |  |  |

^a^ NA: Not available in the literature.

^b^ NS: Not specified in the literature.
