# Supplementary material for: Effect of Calcium and Manganese Supplementation on Heat Resistance of Spores of Bacillus Species Associated With Food Poisoning, Spoilage, and Fermentation
Source: Front Microbiol. 2021 Oct 11;12:744953. doi: 10.3389/fmicb.2021.744953 (PMC8542979; doi:10.3389/fmicb.2021.744953)
Supplement: Supplementary file 10 [file Table_2.DOCX]

**Table S2.** Overview of *D*_100°C_-values for spores of *B. cereus* formed on basal media without mineral supplementation in literature

| Type of strains | Strains used | Pathogenic characteristics | Number of strains | *D*_100°C_ (min) | Sporulation conditions | | |  | Heat treatment condition | Viable cell counting conditions | | | References |
| --- | --- | --- | --- | --- | --- | --- | --- | --- | --- | --- | --- | --- | --- |
|  |  |  |  |  | Media | Temperature (°C) | Time (h) |  | Suspension media | Media | Temperature (°C) | Time (h) |  |
| Type strain | ATCC 14579 | NA ^a^ | 1 | 1.2971 | Trypticase soy agar | 33 | 72 |  | Phosphate buffer | Trypticase soy agar | 33 | 24 | Penna and Moraes (2002) |
|  | ATCC 14579 | NA | 1 | 1 | NA | NA | NA |  | 10 mM KPO_4_ | Diluted nutrient agar | 30 | 72 | De Vries et al. (2004) |
|  | ATCC 14579 | NA | 1 | 0.9 | Cambell's sporulation agar | 35 | within 336 |  | 0.067 M phosphate buffer | Nutrient agar | 35 | NA | Rajkowski and Mikolajcik (1987) |
| Reference strain | T | Emetic | 1 | 1 | G-medium | 30 | 18 |  | 0.05 M potassium phosphate buffer | Trypticase soy agar | NA | NA | Beaman et al. (1982) |
| Isolated strain | R96 | NA | 1 | 6.9 | Cambell's sporulation agar | 35 | within 336 |  | 0.067 M phosphate buffer | Nutrient agar | 35 | NA | Rajkowski and Mikolajcik (1987) |
|  | B_4_ac | Diarrheal | 1 | 2.2 |  |  |  |  |  |  |  |  |  |
|  | B_6_ac | Diarrheal | 1 | 1.7 |  |  |  |  |  |  |  |  |  |
|  | NS ^b^ | NA | 6 | 0.8 | Peptone water | 37 | 336 |  | Peptone water | Plate count agar | 37 | 24 | Janštová et al. (2001) |

^a^ NA: Not available in the literature.

^b^ NS: Not specified in the literature.
