# Supplementary material for: Effect of Calcium and Manganese Supplementation on Heat Resistance of Spores of Bacillus Species Associated With Food Poisoning, Spoilage, and Fermentation
Source: Front Microbiol. 2021 Oct 11;12:744953. doi: 10.3389/fmicb.2021.744953 (PMC8542979; doi:10.3389/fmicb.2021.744953)
Supplement: Supplementary file 11 [file Table_3.DOCX]

**Table S3.** Overview of *D*_100°C_-values for spores of *B. coagulans* formed on basal media without mineral supplementation in literature

| Type of strains | Strains used | Number of strains | *D*_100°C_ (min) | Sporulation conditions | | |  | Heat treatment condition | Viable cell counting conditions | | | References |
| --- | --- | --- | --- | --- | --- | --- | --- | --- | --- | --- | --- | --- |
|  |  |  |  | Media | Temperature (°C) | Time (h) |  | Suspension media | Media | Temperature (°C) | Time (h) |  |
| Type strain | IAM 1194 | 1 | 4.79 | Nutrient agar | 35 | 72 |  | Distilled water | Nutrient agar | 35 | 240 | Nakayama et al. (1996) |
| Reference strain | NRRL B-1103 | 1 | 21 | SMS sporulation medium | 55 | 96 |  | Sorensens buffer | YPTD agar | 55 | 24 | Mallidis et al. (1990) |
|  | NRRL B-1103 | 1 | approximately 3.5 | NA ^a^ | NA | NA |  | Butterfield's phosphate buffer | Glucose yeast extract agar | NA | NA | Majeed et al. (2021) |
| Isolated strain | NS ^b^ | 1 | 1.26 | Peptone water | 37 | 336 |  | Peptone water | Plate count agar | 37 | 24 | Janštová et al. (2001) |
|  | 3105 018 | 1 | 9.3 | Aerobic bacteria medium | 37 | 48-120 |  | 0.2 M phosphate buffer | Aerobic bacteria medium | 37 | 48-120 | André et al. (2013) |

^a^ NA: Not available in the literature.

^b^ NS: Not specified in the literature.
