# Supplementary material for: Effect of Calcium and Manganese Supplementation on Heat Resistance of Spores of Bacillus Species Associated With Food Poisoning, Spoilage, and Fermentation
Source: Front Microbiol. 2021 Oct 11;12:744953. doi: 10.3389/fmicb.2021.744953 (PMC8542979; doi:10.3389/fmicb.2021.744953)
Supplement: Supplementary file 12 [file Table_4.DOCX]

**Table S4.** Overview of *D*_100°C_-values for spores of *B. subtilis* formed on basal media without mineral supplementation in literature

| Type of strains | Strains used | Number of strains | *D*_100°C_ (min) | Sporulation conditions | | |  | Heat treatment condition | Viable cell counting conditions | | | References |
| --- | --- | --- | --- | --- | --- | --- | --- | --- | --- | --- | --- | --- |
|  |  |  |  | Media | Temperature (°C) | Time (h) |  | Suspension media | Media | Temperature (°C) | Time (h) |  |
| Type strain | IAM 12118 | 1 | 0.85 | Nutrient agar | 35 | 72 |  | Distilled water | Nutrient agar | 35 | 240 | Nakayama et al. (1996) |
| Reference strain | CECT 4071 | 1 | 2.56 | Plate count agar | 37 | NA ^a^ |  | Distilled water | Brain heart infusion agar | 37 | 24 | Esteban et al. (2015) |
|  | CECT 4522 | 1 | 1.52 | 2× SG medium | 30 | 24-48 |  | Distilled water | 2× SG medium | 30 | 24-48 | Movahedi et al. (2002) |
| Isolated strain | NS ^b^ | 18 | 1.18 | Peptone water | 37 | 336 |  | Peptone water | Plate count agar | 37 | 24 | Janštová et al. (2001) |
|  | AdHCL | 1 | 6.4 | Plate count agar | 30 | 120 |  | Distilled water | Plate count agar | 30 | 24 | Conesa et al. (2003) |
|  | NS | 1 | 6.2 | 2× SG medium | 30 | 24-48 |  | Distilled water | 2× SG medium | 30 | 24-48 | Movahedi et al. (2002) |

^a^ NA: Not available in the literature.

^b^ NS: Not specified in the literature.
